# An opsonic peptide promotes radiation-induced macrophage extracellular trap clearance to alleviate inflammation

**DOI:** 10.3389/fimmu.2026.1970645

**Published:** 2026-10-12

**Authors:** Yongchan Lee, Gaifeng Ma, Dilara Aylar, Monowar Aziz, Ping Wang

**Affiliations:** 1 Center for Immunology and Inflammation, The Feinstein Institutes for Medical Research, Manhasset, NY, United States; 2 Departments of Surgery and Molecular Medicine, Zucker School of Medicine at Hofstra/Northwell, Manhasset, NY, United States

**Keywords:** extracellular traps, inflammation, integrin, macrophage, METs, OP18, radiation

## Abstract

Ionizing radiation exposure leads to inflammation and tissue damage, in which macrophage extracellular traps (METs) may play a role. Effective strategies to mitigate inflammation caused by radiation-induced METs are needed. We have recently developed a novel opsonic peptide, OP18, that targets multiple damage-associated molecular patterns (DAMPs) and enhances their clearance by macrophages. Because METs contain DAMPs, including histone H3 and high-mobility group box 1 protein (HMGB1), we hypothesize that OP18 promotes the clearance of radiation-induced METs and thereby reduces inflammation. *In vitro* studies were conducted using macrophages exposed to radiation and METs, with or without OP18 treatment. METs uptake, endo-lysosomal trafficking, integrin involvement, mitochondrial function, NF-κB activation, and cytokine release were assessed. OP18 significantly enhances MET uptake by macrophages, an effect that is mediated by α_v_β_3_-integrin and involves the endo-lysosomal trafficking. This enhanced clearance by OP18 alleviates METs-induced mitochondrial stress and restores metabolic function (ATP production, basal/maximal respiration) in macrophages. Furthermore, OP18 treatment exhibits a trend towards decreasing METs-induced NF-κB activation while increasing the release of anti-inflammatory cytokine IL-10. OP18 represents a novel therapeutic approach to mitigate radiation-induced inflammation by promoting the efficient clearance of pro-inflammatory METs, thereby restoring cellular homeostasis and dampening inflammatory signaling.

## Introduction

Life-threatening ionizing radiation can result from catastrophic events such as nuclear detonations, severe reactor or criticality accidents, unshielded industrial radiation sources, radiotherapy misadministration, and extreme solar particle events in space (1). Within living tissues, ionizing radiation exerts its detrimental effects by disrupting chemical bonds within molecules. This molecular disruption primarily damages cellular processes and, critically, DNA structure, forming the basis of its profound impact on human health (2). Severely damaged cells may consequently undergo programmed cell death (apoptosis) or necrosis (3). When many cells within a tissue or organ are damaged or die, its function becomes impaired, leading to severe clinical manifestations including skin burns, gastrointestinal distress, myelosuppression, and multi-organ failure (4, 5). A critical consequence of ionizing radiation exposure is profound immune suppression, stemming from the damage and depletion of highly radiosensitive immune cells, particularly lymphocytes and their bone marrow precursors. This compromised immunity significantly diminishes the host’s ability to combat infections and impedes effective tissue repair and immune surveillance. Considering these severe and multifaceted pathological effects, medical countermeasures are vital to rapidly mitigate radiation injury, prevent complications, and improve survival outcomes (6).

Ionizing radiation profoundly compromises macrophage function and viability through generation of deleterious free radicals. This damage consequently leads to critical macrophage dysfunctions, including impaired phagocytosis, altered cytokine production (frequently skewed towards a pro-inflammatory or pro-fibrotic phenotype), and a modulated capacity for antigen presentation (7, 8). Macrophage extracellular traps (METs) are reticular structures expelled by macrophages into the extracellular space, primarily comprising decondensed chromatin (DNA and associated histones) along with various antimicrobial proteins and enzymes (9). Analogous to neutrophil extracellular traps (NETs), the primary function of METs involves physically ensnaring and concentrating pathogens (e.g., bacteria, fungi, viruses, and parasites), thereby preventing their dissemination and facilitating their neutralization or elimination by embedded proteins (9). Consequently, MET formation holds significant clinical relevance across a range of pathologies, including autoimmune diseases, cardiovascular disorders, cancer, infectious diseases, chronic inflammatory conditions, and neurological disorders (10). While the induction of NETs by radiation is known (11, 12), recent evidence demonstrates that METs are also formed in response to ionizing radiation, where they promote inflammation and worsen tissue injury (13). Current anti-inflammatory strategies targeting extracellular traps (ETs) primarily focus on inhibiting their formation (14), degrading existing structures (e.g., with DNase I) (15), or neutralizing pathogenic components (16). However, these methods often present limitations, including off-target effects, short systemic half-lives, or the potential to compromise beneficial immune responses. Consequently, a significant unmet need exists for novel, highly specific, and effective interventions capable of mitigating MET-driven inflammation, particularly those employing multi-modal mechanisms like enhanced biological clearance.

Damage-associated molecular patterns (DAMPs) bind TLR4, amplifying inflammation, organ injury, and worsening outcomes following exposure to radiation (17–19). Targeting DAMPs therefore represents a promising therapeutic strategy for radiation injury. We developed Opsonic Peptide 18 (OP18), a novel 18-amino acid (aa) peptide designed to simultaneously target major DAMPs such as extracellular cold-inducible RNA-binding protein (eCIRP), high mobility group box-1 protein (HMGB1), and histone H3 (20, 21). Protein interaction modeling revealed a shared 15-aa extracellular TLR4 binding region among these DAMPs. An RGD motif is incorporated into OP18 to engage the α_v_β_3_-integrin on phagocytes. Thus, OP18 sequesters DAMPs and facilitates their α_v_β_3_-integrin-dependent macrophage uptake, thereby resolving inflammation in sepsis. METs contain histone H3 (22). Recent studies also revealed the presence of HMGB1 on extracellular traps (23). We therefore hypothesize that OP18 can target DAMPs present in the METs (20), thereby facilitating their clearance by macrophages. Thus, OP18 represents a promising therapeutic candidate for mitigating MET-driven inflammation and cellular stress in radiation injury.

## Materials and methods

### Experimental animals

Male C57BL/6 wild-type mice, aged 8 to 12 weeks, were procured from The Jackson Laboratory (Bar Harbor, ME). Mice were maintained in a temperature-controlled vivarium with a 12-h light/dark cycle and provided *ad libitum* access to standard rodent chow and water. All animal experiments adhered to the National Institutes of Health (NIH) guidelines for the care and use of laboratory animals. Animal study protocols were approved by the Institutional Animal Care and Use Committee (IACUC) of the Feinstein Institutes for Medical Research.

### Reagents

Hoechst 33342 (H33342) (cat. no. R37605), SYTOX Orange (cat. no. S11368), mitotracker Green FM (cat. No. M7514), and mitotracker Red CMXRos (cat. No. M7512) were purchased from Thermofisher Scientific (Waltham, MA). Antibodies such as anti-CD63 Abs (Cat. No. 217345), Alexa Fluor 647 anti-LAMP1 Abs (Cat. No. ab237307), and anti-histone H3 (citrulline R2 + R8 + R17) Abs (Cat. No. ab281584) were purchased from Abcam (Waltham, MA). Antibody against α_v_β_3_-integrin (Cat. No. BS-1310R) was purchased from Bioss (Woburn, MA). Antibody against NF-κB p65 (Cat. No. 622601) was purchased from Biolegend. Small chemical inhibitors: Src family kinase inhibitor, Dasatinib (final concentration, 1 μM, Cat. No. HY-10181), and focal adhesion kinase inhibitor, PF573228 (final concentration, 10 μM, Cat. No. HY-10461) were purchased from MedChemExpress (Monmouth junction, NJ). ROCK inhibitor, Y-27632 (final concentration, 10 μM, Cat. No. S1049) was purchased from Selleckchem (Houston, TX).

### Isolation and culture of peritoneal macrophages

Mouse euthanasia was performed by introducing compressed carbon dioxide gas into an uncharged, clear chamber. Peritoneal washout cells were collected from mice via peritoneal lavage. Lavage was performed by flushing the mouse peritoneum with 10 mL of cold phosphate-buffered saline (PBS) supplemented with 2% fetal bovine serum (FBS). The collected cells were then precipitated by centrifugation and subsequently seeded into cell culture vessels. Routine ell number counting gives about five million cells per mouse. Cells were maintained in RPMI medium supplemented with 10% FBS and penicillin–streptomycin. Following overnight incubation, non-adherent cells were removed by washing the culture vessels three times with fresh cell culture medium.

### X-ray irradiation

Non-adherent peritoneal cells were removed after overnight culture. Adherent peritoneal cells were irradiated with 10-Gy (3-Gy/min) using an X-ray Precision X-Rad320 (Precision X-Ray Inc., Madison, CT) (24).

### METs preparation

The DNA preparation was done with a modified method of Halder et al. (25). Extracellular DNA was collected from the culture supernatants of peritoneal macrophage after 24 h incubation post-treatment of 10-Gy radiation in the presence of SYTOX Orange. The bottom of each well of the plate was vigorously flushed 5 times with micropipette. The culture media were subjected to centrifuge, 400×*g* for 10 min at 4 °C. The cell pellets were discarded, and the supernatants were immediately subjected to centrifuge, 12,000×*g* for 20 min at 4 °C. The supernatant was discarded, and the pellet was resuspended in sterile PBS. DNA quantification was performed with Qubit high sensitivity DNA quantification kit (Thermo Fisher Scientific).

### Immunofluorescence staining

Peritoneal cells were seeded at a density of 1 × 10^6^ cells per 35-mm glass-bottom dish (Cat. No.: P35G-1.5-20-C, Mattek, Ashland, MA) and cultured overnight. Following overnight culture, non-adherent cells were removed by washing, and the remaining adherent cells were then subjected to experimental treatments. Cells were fixed with 4% paraformaldehyde (PFA) for 15 minutes at room temperature, followed by three washes with phosphate-buffered saline (PBS). Fixed cells were subsequently blocked for 2 h with a solution of 5% bovine serum albumin (BSA) and 100 mM glycine in PBS, prior to overnight incubation at 4 °C with the primary antibody diluted in this blocking solution. After three PBS washes, cells were incubated with the fluorescently conjugated secondary antibody for 2 h at room temperature. Following another three washes with PBS, immunofluorescence specimens were mounted using ProLong Gold Antifade (Cat. No.: P36934, ThermoFisher Scientific).

### Confocal microscopy

High-resolution single cell images were acquired using a Zeiss LSM 900 confocal microscopy system mounted on an Axio Observer.Z1/7 inverted microscope. Z-stack images of cells were captured with a Plan-Apochromat 63×/1.40 Oil DIC M27 objective lens. Images were acquired employing the SR-4Y fast acquisition mode of Airyscan, utilizing four times averaging. Acquired confocal images were subsequently processed and merged using FIJI ImageJ software (26). Live confocal imaging for Mitotracker Green and Red was done with a Plan-Apochromat 20×/0.8 M27 objective lens.

### Quantitative image analysis

Confocal images with immunofluorescence staining were analyzed with FIJI ImageJ. Low magnification with Plan-Apochromat Plan-Apochromat 20X/0.8 M27 objective was used for imaging. Image acquisition was done with 2X2 tiling and stitched by Zeiss Zen blue software. Typically, the analysis was done with over 200 cells per stitched image for the quantification. The average of cell quantification per each image was represented as an individual data point in each graph presented. Nuclear counting was done with Hoechst33342 image. Fluorescence quantification was done with thresholding and segmentation by making binary mask. To measure SYTOX Orange signal only in the METs area the signal in the nuclear region was removed by applying region of interest (ROI) of nuclear binary mask and filing process. Then, the METs region was counted by generating a binary mask with thresholding command. The resulting METs puncta count was normalized by the number of nuclei in the field. For the quantification of Mitotracker green and red fluorescence, single cell ROIs were generated from the binary mask of Mitotracker green image, and they were used for measuring fluorescence intensity of both Mitotracker dyes. For quantitative analysis of NF- κB p65 fluorescence intensity in whole cell and nuclear area of single cell, we have made ROIs for each single cell area and nuclear area and measure the fluorescence intensity for whole cell NF-κB and nuclear NF-κB. For analysis of large number of cells in whole microscopic field, we used histogram analysis for identifying cells having higher signal of NF-κB.

### Mitochondrial oxygen consumption rate (OCR) assay

Mitochondrial oxygen consumption rate (OCR) in peritoneal macrophages was quantified using a Seahorse XF wave and XF Pro software (Agilent, North Billerica, MA) in conjunction with the Mito Stress Test Kit (Cat. No. 103015-100, Agilent, North Billerica, MA). Peritoneal macrophages were seeded at 1 × 10^5^ cells/well in Seahorse microplates and treated with METs (20 ng/mL) in the presence or absence of OP18 (2 µg/mL). The treatment was done in Seahorse RPMI without phenol red with 2 mM glutamine, 10 mM glucose and 1 mM pyruvate, PH7.4. Assays were performed 4 h post-treatment. The assay protocol followed sequential injections of specific modulators: oligomycin (1.5 µM), carbonyl cyanide-4 (trifluoromethoxy) phenylhydrazone (FCCP—1 µM), and a mixture of rotenone and antimycin A (0.5 Um). Oligomycin, an ATP synthase inhibitor, was injected first into the assay following basal measurements. It decreases electron flow through the ETC, resulting in a reduction in mitochondria respiration. This decrease in OCR is linked to determine cellular ATP production. FCCP, an uncoupler agent, was a second injection, to collapse the proton gradient and disrupt the mitochondria membrane potential. The FCCP-stimulated OCR can be used to assess maximal respiration and spare respiratory capacity. Finally, rotenone (complex I inhibitor) and antimycin A (complex III inhibitor) were added to inhibit mitochondrial respiration, thereby quantifying non-mitochondrial oxygen consumption.

### Enzyme-linked immunosorbent assay (ELISA)

Isolated mouse peritoneal macrophages were cultured and subsequently treated with METs (20 ng/mL) in the presence or absence of OP18 (2 µg/mL). Twenty-four hours post-treatment, cell culture supernatants were collected to quantify IL-6 and IL-10 levels using ELISA kits (IL-6: Cat. No. 555240; IL-10: Cat. No. 555252, BD Biosciences, Franklin Lakes, NJ), strictly following the manufacturer’s instructions. Recombinant mouse IL-6 and IL-10 standards were used to establish standard curves for cytokine quantification. Briefly, 100 µL aliquots of samples or serially diluted cytokine standards were loaded into wells of the capture antibody-precoated plates. The plates were then incubated for 2 h at RT, followed by washing. After washing, biotinylated detection antibody and HRP-conjugated Streptavidin were added and incubated for 1 h at RT. After incubation with a chromogenic substrate, the enzymatic reaction was stopped, and optical density was measured at 450 nm using a BioTek Synergy Neo2 multimode reader (BioTek).

### Statistical analysis

All data are presented as mean ± standard error of the mean (SEM). Statistical comparisons between two groups were performed using a two-tailed Student’s *t*-test, while one-way ANOVA with a *post-hoc* Tukey test was employed for multiple-group comparisons. Statistical significance was defined as a p-value of ≤0.05. Data analysis and graphical representations were generated using GraphPad Prism software (GraphPad Software v.10.2.0, San Diego, CA).

## Results

### OP18 enhances radiation-induced METs clearance by macrophages via α_v_β_3_-integrin

We previously showed that ionizing radiation caused macrophage phagocytic dysfunction (8, 27). To determine if OP18 could enhance the clearance of METs by irradiated macrophages, we subjected macrophages to 10-Gy irradiation. Subsequently, these cells were treated with exogenous, radiation-induced METs (pre-stained with SYTOX Orange) in the presence or absence of OP18 and a neutralizing anti-α_v_β_3_-integrin antibody. METs clearance was quantified by counting SYTOX Orange-positive puncta per cell in the microscopic field. DNA puncta refers to small, distinct dots or localized clusters of DNA signals visible inside the cell when viewed under a microscope. Our data showed that while normal (non-irradiated) macrophages readily internalized METs (**Supplementary Figures 1A, B**), OP18 treatment significantly enhanced their uptake (**Supplementary Figures 1C–E**). While METs alone did not significantly increase the number of puncta, OP18 significantly enhanced SYTOX Orange-positive puncta per cell by 3.6-fold in 10-Gy irradiated macrophages. This OP18-induced enhancement was significantly abrogated by co-treatment with the α_v_β_3_-integrin antibody (**Figures 1A, B**). These results suggest that OP18 enhances the uptake or clearance of METs by irradiated macrophages, and this effect is mediated by α_v_β_3_-integrin.

**Figure 1:**
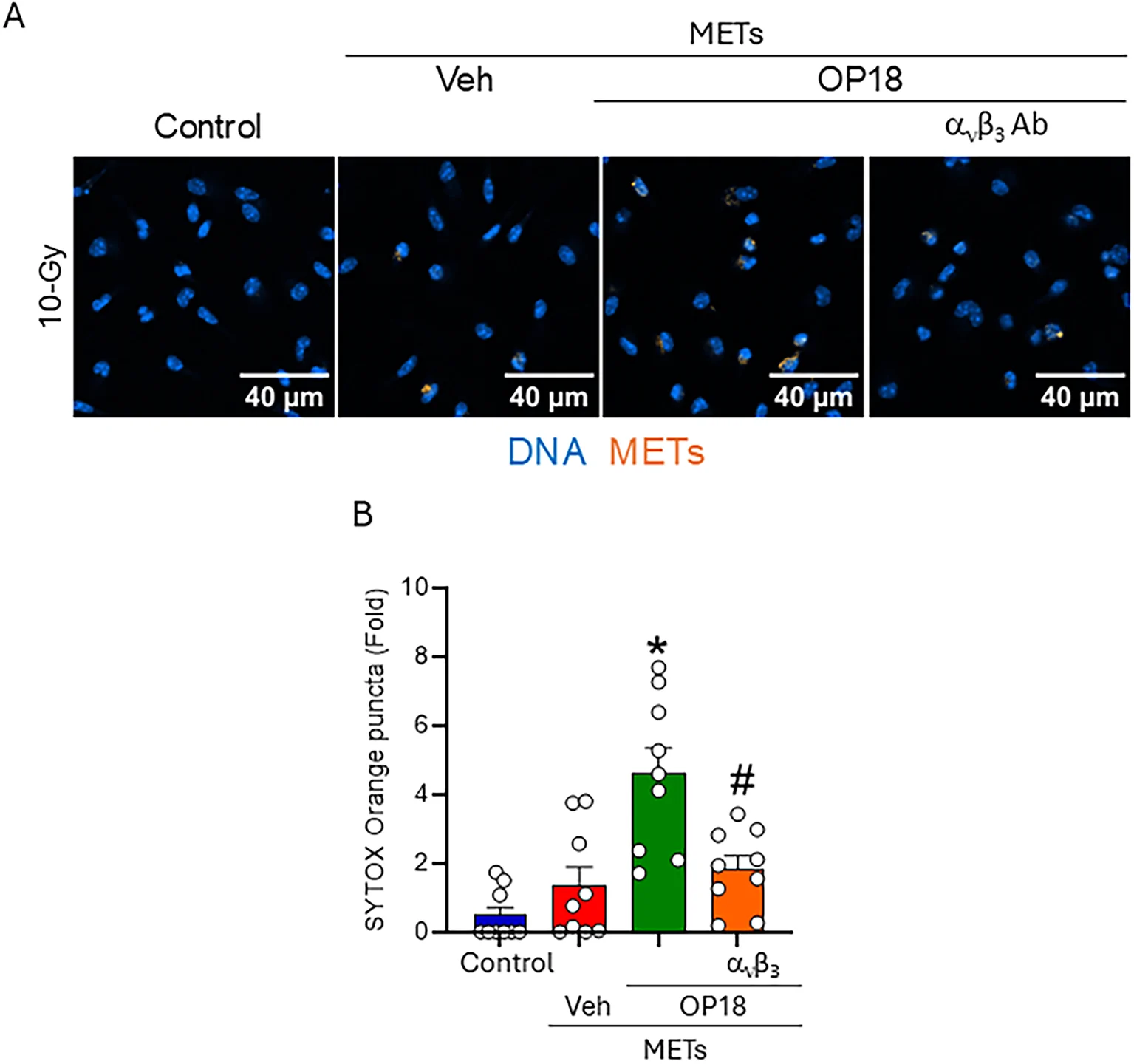
OP18 enhances radiation-induced extracellular trap clearance by macrophages. **(A)** Confocal microscopy images demonstrate the uptake of macrophage extracellular traps (METs) in macrophages following radiation exposure and OP18 treatment. Quantification of SYTOX Orange-positive METs puncta per cell following various treatments. Peritoneal macrophages were irradiated with either 10-Gy, incubated for 3 h, and subsequently treated with exogenous radiation-induced METs (20 ng/mL) for 24 h. Treatments included: Control (no METs), METs alone, METs with OP18 (2 µg/mL), and METs with OP18 and a neutralizing α_v_β_3_ antibody (α_v_β_3_ Ab). **(B)** The number of SYTOX Orange-positive puncta per cell, representing METs uptake or association, was quantified. At 10-Gy, compared to METs treatment alone, OP18 significantly increased the number of SYTOX Orange-positive puncta per 100 cells (*p < 0.05 vs. METs alone). This OP18-mediated enhancement was significantly reversed by co-treatment with the neutralizing α-α_v_β_3_ antibody (^#^p < 0.05 vs. METs + OP18). The data is representative of two biologically independent experiments. Replicates in each group are the average value of cell quantification (cell number > 200) from 9 different image fields. Data are presented as mean ± SEM. Statistical significance was determined by one-way ANOVA with *post-hoc* Tukey test.

### OP18 enhances clearance of METs via endo-lysosomal trafficking

To show intracellular trafficking of the internalized METs in the irradiated macrophages we performed immunofluorescence assays. Confocal microscopy showed evidence demonstrating that the endo-lysosomal trafficking of METs within the irradiated macrophages. Macrophages were irradiated with 10-Gy, treated with exogenous METs and OP18, and then stained for endo-lysosomal marker (CD63 and LAMP1), DNA, and citrullinated histone H3 (citH3, a METs component). Representative confocal images of macrophages stained for LAMP1 (lysosomal marker, Red), DNA (blue), CD63 (endosomal/lysosomal marker, green), and METs (SYTOX Orange). There is a co-localization of METs (SYTOX Orange) with CD63, indicating that internalized METs are transported via CD63-positive endo-lysosomal compartments within the irradiated macrophages. (**Figure 2A**). As a preliminary study, co-localization of METs and CD63 in non-irradiated peritoneal macrophages was also tested. These macrophages showed clear co-localization of METs and CD63 at 24 h after the treatment (**Supplementary Figure 2A**). Furthermore, in cells treated with 10-Gy and METs, there is visible uptake of METs in the merged images with a noticeable co-localization between METs (citH3/SYTOX Orange) and LAMP1 (**Figure 2B**), using a similar setup with LAMP1 (lysosomal marker, Red), DNA (blue), citH3 (METs component, green), and METs (SYTOX Orange, indicating extracellular DNA). As a similar approach using non-irradiated macrophages, the macrophage showed clear co-localization of METs, citH3, and LAMP1 at 24 h after the treatment (**Supplementary Figure 2B**). The data suggest that cells direct ingested METs towards the endo-lysosomal compartment.

**Figure 2:**
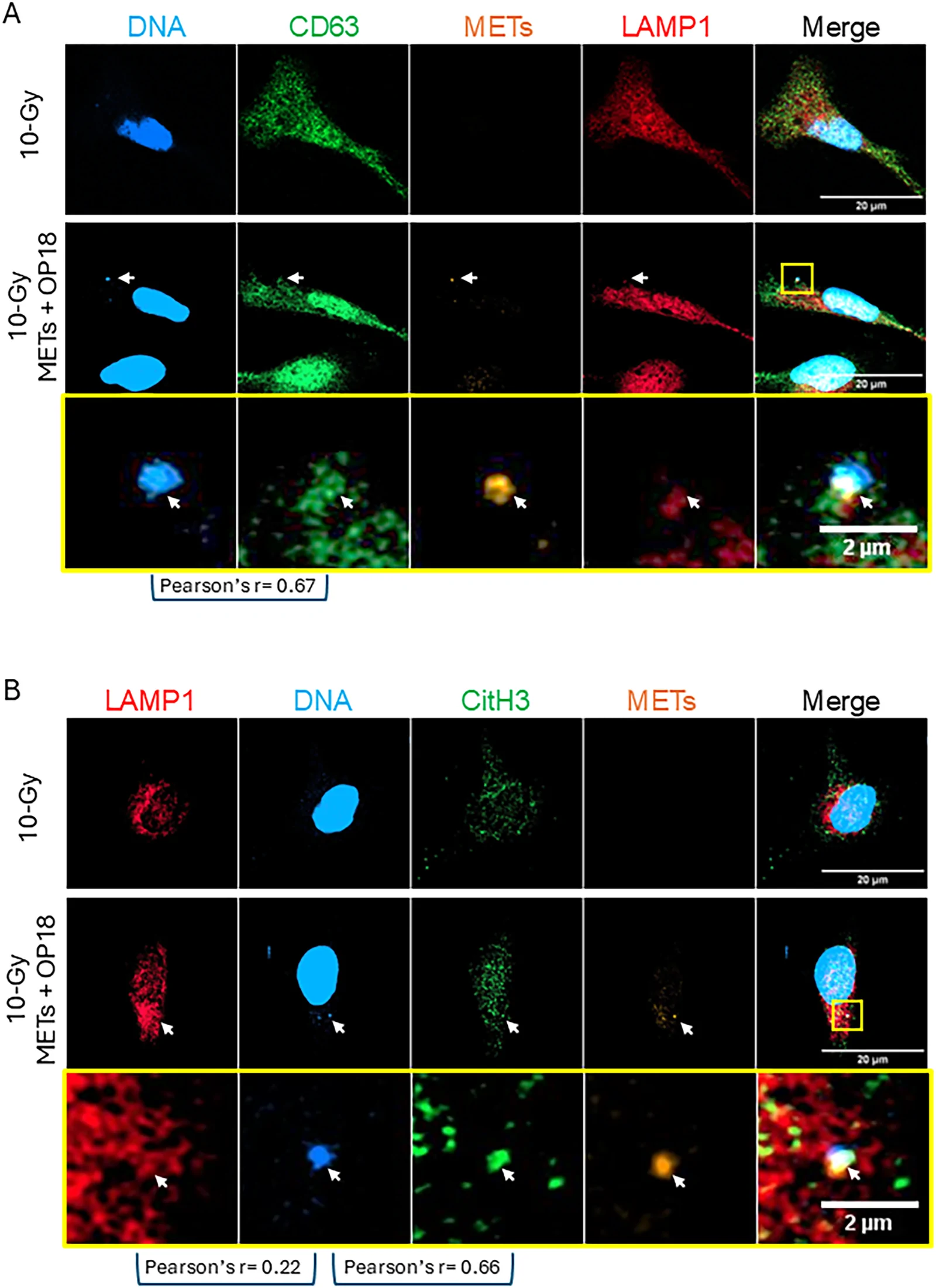
OP18 enhances clearance of macrophage extracellular traps (METs) via endo-lysosomal trafficking. Confocal microscopy images demonstrate the endo-lysosomal trafficking of exogenous radiation-induced METs in macrophages following radiation exposure and OP18 treatment. Macrophages were irradiated with 10-Gy, treated with exogenous METs (20 ng/mL) with or without OP18 (2 µg/mL), and subsequently fixed and stained. **(A)** Images show co-localization of DNA (blue), CD63 (endosomal marker, green), SYTOX Orange (METs DNA, orange), and LAMP1 (lysosomal marker, red) in 10-Gy irradiated macrophages treated with or without METs + OP18. Arrowheads indicate the ingested METs puncta in the cell. **(B)** images show co-localization of LAMP1 (red), DNA (blue), citH3 (METs components, green), and SYTOX Orange (METs DNA, orange) under same experimental setting with panel **(A)**. Arrowheads indicate the ingested METs puncta in the cell. Quantification of co-localization between intracellular markers and METs was assessed with Pearson’s correlation coefficient (r).

### OP18 enhances clearance of METs via α_v_β_3_-integrin-mediated downstream pathway

As shown that the OP18 significantly increased METs ingestion and, it was reversed by the treatment of anti-integrin antibody, we explored the role of integrins, specifically α_v_β_3_, in the OP18-mediated clearance of METs by the irradiated macrophages. Macrophages were treated with exogenous METs (and/or OP18) with or without prior irradiation and stained for DNA and α_v_β_3_-integrin by anti-α_v_β_3_-integrin antibody. Representative confocal images demonstrate the co-localization of DNA (blue), α_v_β_3_-integrin (green), and METs (orange) in the irradiated macrophages treated with METs alone or METs + OP18 (**Figure 3A**). In the METs + OP18 group, there is a visually enhanced co-localization between METs and α_v_β_3_-integrin in internalized vesicles. Quantitatively, Pearson’s correlation coefficient (r) analysis of the colocalization suggests the bridging interaction of histone on METs, OP18, and integrin on the surface of the irradiated macrophages (**Figure 3B**). The median r values for METs alone and METs + OP18 were -0.024 and 0.4085, respectively. Consistent with irradiated macrophages, non-irradiated macrophages METs + OP18 group displays increased apparent co-localization of METs with α_v_β_3_-integrin compared to METs alone group (**Figure 3C**). Pearson’s correlation coefficients (r) for α_v_β_3_-integrin and METs in non-irradiated cells also showed significant increase of positive correlation of co-localization of METs with α_v_β_3_-integrin (**Figure 3D**). The median r values for METs alone and METs + OP18 were 0.1125 and 0.3600, respectively. These findings indicate that OP18 enhances the association and internalization of METs via α_v_β_3_-integrin.

**Figure 3:**
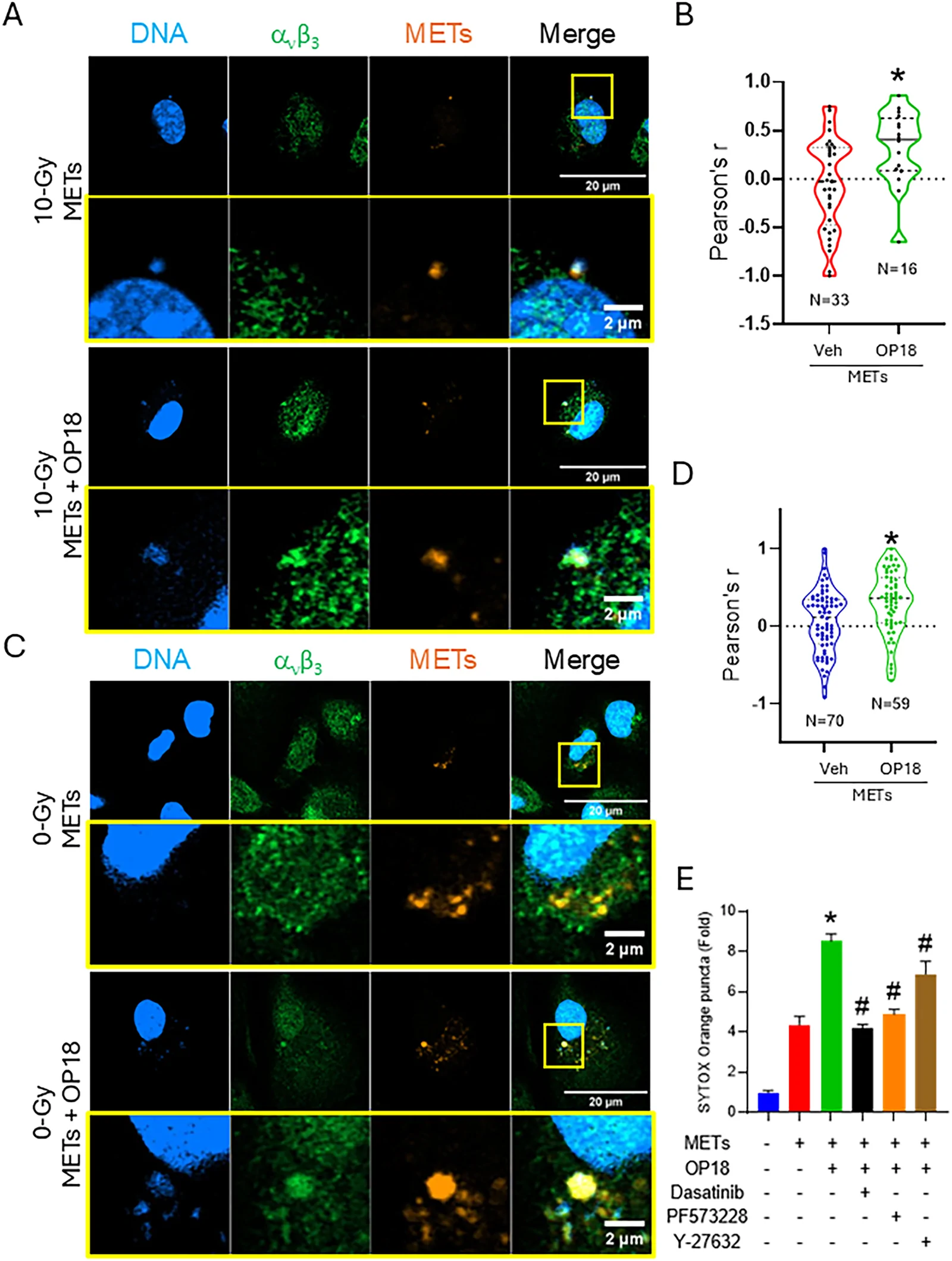
OP18 enhances clearance of macrophage extracellular traps (METs) via integrin-mediated endocytosis. Macrophages were either **(A, C)** irradiated with 10-Gy or **(B, D)** non-irradiated (0-Gy), then treated with exogenous METs (20 ng/mL) with or without OP18 (2 µg/mL). Cells were fixed and stained for DNA (blue), α_v_β_3_-integrin (green), and METs (orange). **(A, C)** Representative confocal images illustrating the co-localization of DNA, α_v_β_3_-integrin, and METs in the indicated treatment groups. **(B, D)** Quantification of co-localization between α_v_β_3_-integrin and METs was done using Pearson’s correlation coefficient (r) for 10-Gy irradiated and non-irradiated macrophages, respectively (both panels: *p < 0.05 vs. METs). 0-Gy and 10-Gy treatment were performed in two biologically independent experiments. Data are presented as mean ± SEM. Statistical significance was determined by Student’s *t*-test. **(E)** The fold increase of SYTOX Orange-positive puncta, representing METs uptake or association, was quantified in normal macrophages with the treatment of various pharmacological inhibitors. At 24 h after treatment, METs uptake was compared to METs treatment alone, OP18 significantly increased the number of SYTOX Orange-positive puncta (*p < 0.05 vs. METs alone). This OP18-mediated enhancement was significantly reversed by co-treatment with dasatinib, PF573228, and Y-27632 (all groups: ^#^p < 0.05 vs. METs + OP18). Replicates in each group are the average value of cell quantification (cell number > 200) from 9 different image fields. Data are presented as mean ± SEM. Statistical significance was determined by one-way ANOVA with *post-hoc* Tukey test.

To identify the underlying mechanism of METs uptake by OP18 we tested intracellular signaling events in non-irradiated macrophages by employing pharmacological inhibitors against Src family kinase (dasatinib), focal adhesion kinase (PF573228), and ROCK (Y-27632) (**Figure 3E**). Dasatinib and PF573228 apparently reversed the ability of OP18 in METs uptake of macrophages, whereas Y-27632, which is not directly targeting downstream component of integrin receptor signaling, was less effective in the inhibition of OP18 effect on the METs uptake (**Figure 3E**). Considering the immediate involvement of Src family kinase and focal adhesion kinase in the outside-in signaling of integrin receptor on the cell surface the result suggests that the OP18-mediated endocytosis is governed by outside-in signaling of the integrin receptor on the cell surface.

### OP18 alleviates METs-induced mitochondrial stress

Mitochondrial function of macrophages was assessed by measuring real-time oxygen consumption rate (OCR) traces for control, METs, and METs + OP18 groups, illustrating the dynamic changes in mitochondrial respiration after sequential additions of oligomycin, FCCP, and rotenone/antimycin A (**Figure 4A**). METs exposure significantly decreased mitochondrial ATP production, maximal respiration, and basal respiration (**Figures 4B–D**). In all three parameters, co-treatment with OP18 significantly reversed these METs-induced reductions, bringing the OCR parameters closer to control values. Mitochondrial potential was assessed using MitoTracker Red CMXRos (reflecting mitochondrial membrane potential) and MitoTracker Green (reflecting mitochondrial mass) (**Figure 4E**). Non-irradiated macrophages were subjected to the treatment of METs and stained with both of Mitotrackers at the same time. Confocal images of macrophages were acquired with MitoTracker Green and Red CMXRos for Control, METs, and METs + OP18 groups. Representative images show marked decrease in red fluorescence signal for cells exposed to METs alone. Corresponding bar graph quantifies the Red MFI/Green MFI ratio in each cell (**Figures 4F, G**). Both quantitative measurements at 6- and 24-h after treatment of METs showed significant decrease in the ratio of mitochondrial potential over mitochondrial mass in cells. METs exposure significantly reduced this ratio, indicating mitochondrial depolarization. Treatment with OP18 rescued this reduction, restoring the ratio closer to control levels. Together, these data indicate that OP18 effectively protects macrophages from METs-induced mitochondrial stress and dysfunction.

**Figure 4:**
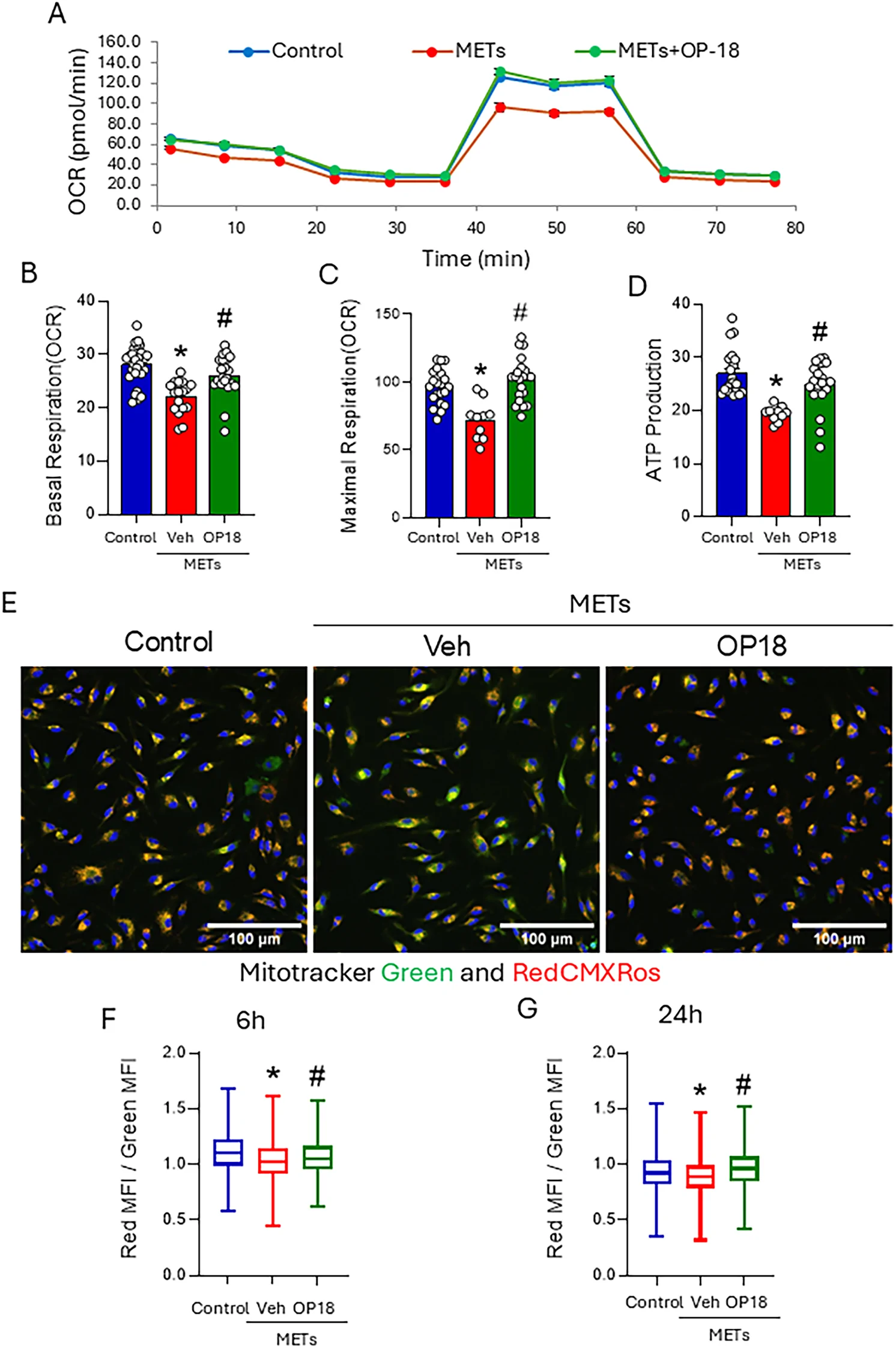
OP18 alleviates mitochondrial stress induced by macrophage extracellular traps (METs) exposure. Normal peritoneal macrophages were treated with Control (PBS), METs (20 ng/mL), or METs + OP18 (2 µg/mL). **(A)** Representative real-time oxygen consumption rate (OCR) traces from a Seahorse XF Mito Stress Test for Control, METs, and METs + OP18 treated cells. Sequential injections of oligomycin, FCCP, and rotenone/antimycin A are indicated. **(B-D)** Quantification of key mitochondrial respiration parameters derived from the Seahorse assay: Basal Respiration, Maximal Respiration, and ATP Production. METs significantly decreased all three parameters (*p < 0.05 vs. Control), and OP18 significantly reversed these reductions (^#^p < 0.05 vs. METs). Data are presented as mean ± SEM. Statistical significance was determined by one-way ANOVA with *post-hoc* Tukey test. The data is representative of two biologically independent experiments. **(E)** Representative confocal images of macrophages stained with MitoTracker Green (mitochondrial mass) and MitoTracker Red CMXRos (mitochondrial membrane potential). **(F, G)** Quantification of the ratio of MitoTracker Red MFI/Green MFI at 6 and 24 h after the treatment, indicating mitochondrial polarization. METs significantly reduced this ratio (*p < 0.05 vs. Control), which was significantly rescued by OP18 (#p < 0.05 vs. METs). Data are presented as mean ± SEM. Statistical significance was determined by one-way ANOVA with *post-hoc* Tukey test.

### OP18 suppresses METs-induced NF-κB activation

The effect of METs on NF-κB activation, a key pro-inflammatory signaling pathway, in macrophages was investigated. Macrophages were treated with exogenous METs and stained for DNA, NF-κB (p65 antibody), and SYTOX Orange (METs). Representative confocal images (**Figure 5A**) illustrate the localization of NF-κB (green), DNA (blue), and SYTOX Orange (red) in Control and METs groups. In the METs-treated cells, there is a visible increase in nuclear NF-κB staining compared to control, suggesting NF-κB activation and translocation. Quantitative analysis of NF-κB fluorescence intensity in whole cell and nuclear compartment was done with 3 untreated control cells vs. 3 METs ingested cells, having SYTOX Orange positive puncta (**Figures 5B–D**). METs exposure significantly increased the whole cell MFI of NF-κB. Furthermore, METs significantly increased the Nuclear MFI and the Nuclear MFI/Whole cell MFI ratio, confirming increased NF-κB activation and nuclear translocation. METs treatment significantly increased both the percentage of cells with high whole cell NF-κB fluorescence (**Figures 5E, F**) and the percentage of cells with high nuclear NF-κB fluorescence (**Figures 5G, H**). OP18 treatment appeared to reduce these percentages, though statistical significance is not indicated for the METs vs. METs + OP18 comparison. Collectively, these findings suggest that OP18 treatment showed a trend toward decreasing MET-induced NF-κB activation and may modulate this signaling pathway.

**Figure 5:**
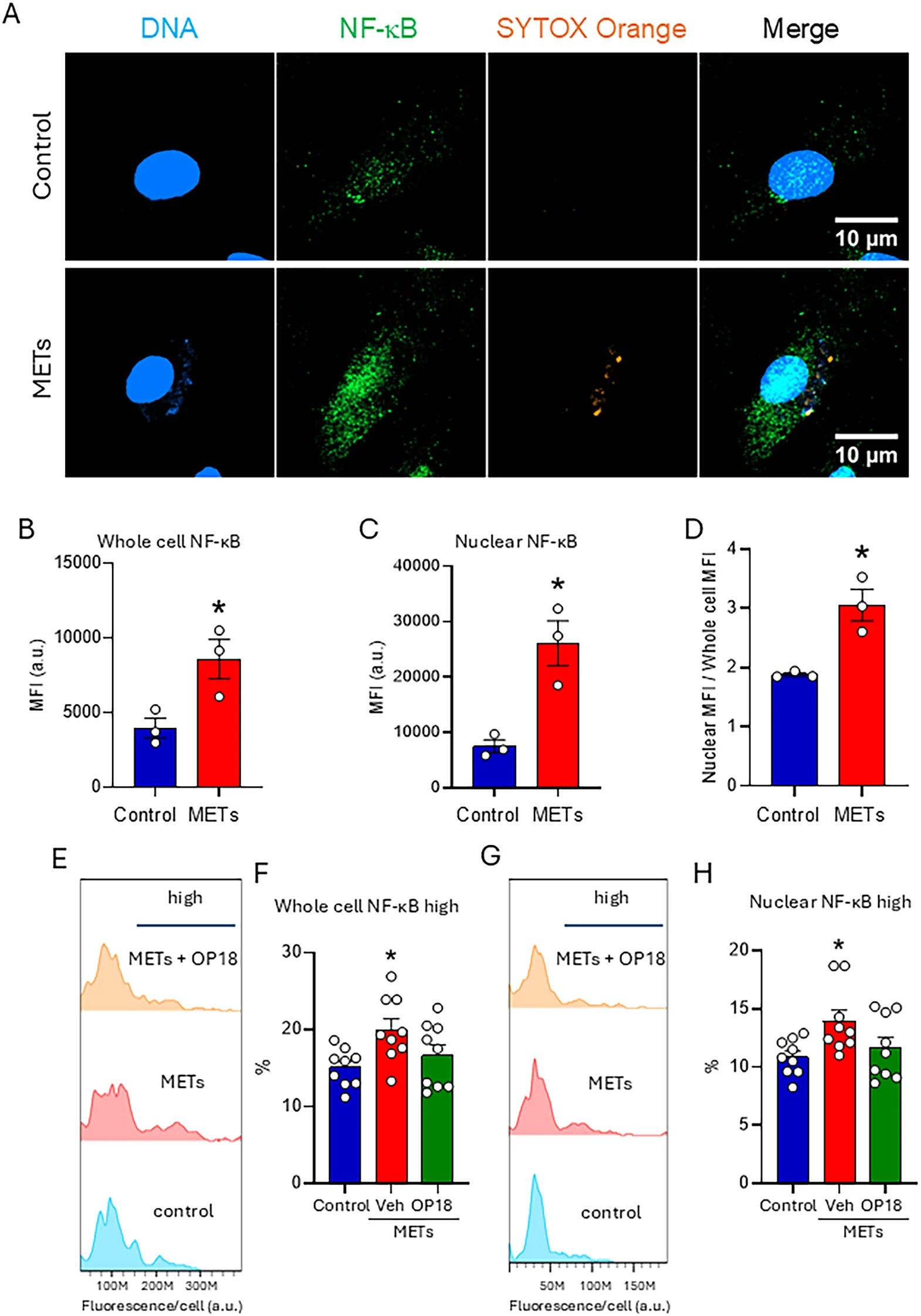
OP18 suppresses macrophage extracellular trap (METs)-induced NF-κB activation. Peritoneal macrophages were treated with Control, METs (20 ng/mL), or METs + OP18 (2 µg/mL), then fixed and immunofluorescently stained for NF-κB p65. **(A)** Representative confocal image of three single cell confocal images showing DNA (blue), NF-κB (green), and SYTOX Orange (orange) localization. **(B-D)** Quantification of whole cell mean fluorescence intensity (MFI) of NF-κB (left), nuclear MFI of NF-κB (middle), and ratio of nuclear MFI to whole cell MFI for NF-κB (right). **(E, F)** Percentage of cells exhibiting high whole cell NF-κB fluorescence and **(G, H)** high nuclear NF-κB fluorescence. METs significantly increased NF-κB activation and nuclear translocation across all quantified parameters (*p < 0.05 vs. Control). OP18 treatment showed a tendency to reduce these METs-induced increases, although specific statistical significance is not indicated between METs and METs + OP18 groups (cytosolic: p = 0.15 vs. METs, nuclear: p = 0.15 vs. METs). Data are presented as mean ± SEM. Statistical significance was determined by one-way ANOVA with *post-hoc* Tukey test.

### OP18 induces anti-inflammatory cytokine secretion

The impact of OP18 on the cytokine production profile of macrophages exposed to METs was examined. The measurement was done for pro-inflammatory cytokine IL-6 and the anti-inflammatory cytokine IL-10. Macrophages were treated with exogenous METs (and/or OP18), and cell culture supernatants were collected at 1-day post-treatment for ELISA quantification of IL-6 and IL-10. The levels of IL-6 in the cell culture supernatants of METs treatment significantly increased compared to the control group (**Figure 6A**). While OP18 co-treatment with METs doesn’t reduce significantly IL-6 levels compared to METs alone, which statistical significance is not indicated for the METs vs. METs + OP18 comparison (p = 0.18), for IL-10 levels, METs treatment did not significantly alter IL-10 production compared to control (p = 0.15) (**Figure 6B**). OP18 treatment with METs significantly increased the production of IL-10. These results suggest that METs induce a pro-inflammatory response characterized by elevated IL-6, and OP18 may mitigate this IL-6 increase, while it enhances anti-inflammatory response by increased IL-10.

**Figure 6:**
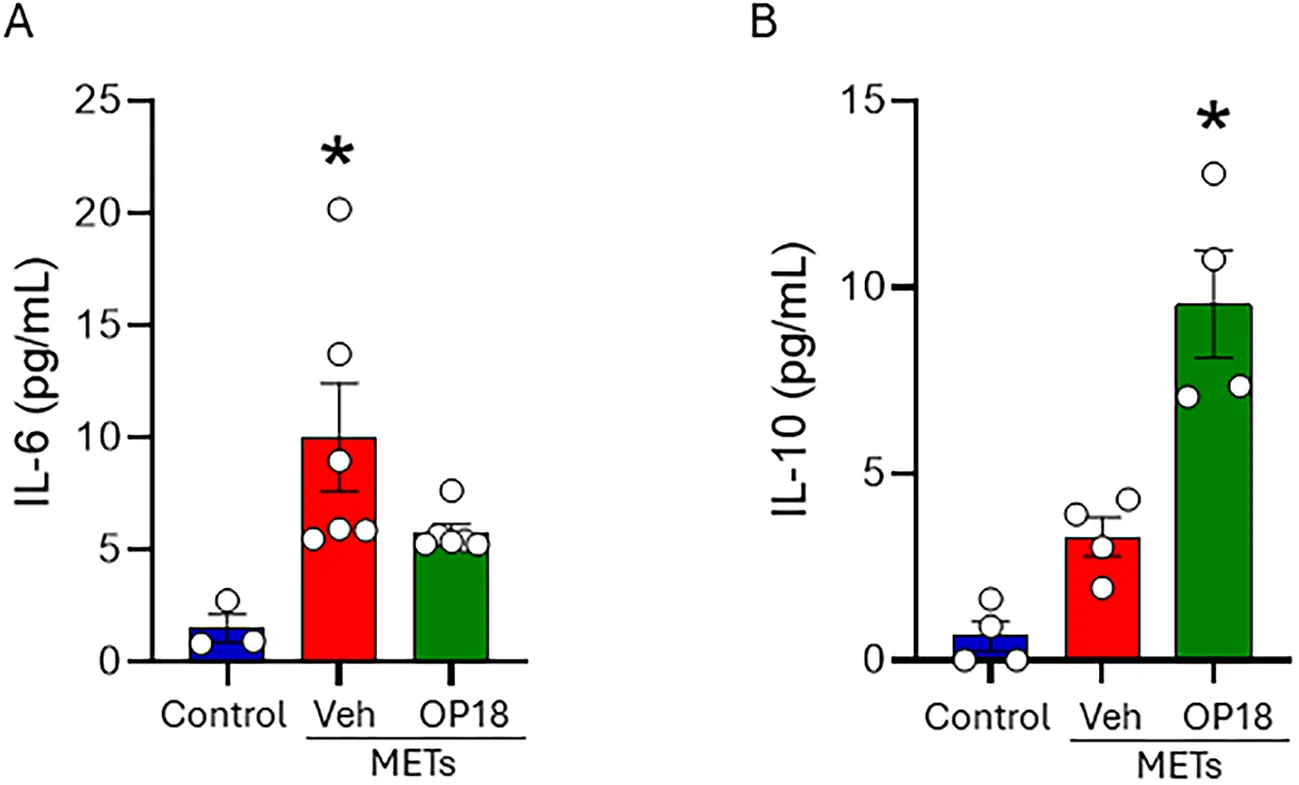
OP18 suppresses macrophage extracellular trap (METs)-induced pro-inflammatory cytokine production. Peritoneal macrophages were treated with Control, METs (20 ng/mL), or METs + OP18 (2 µg/mL). Supernatants were collected for 1-day post-treatment. Enzyme-linked immunosorbent assay (ELISA) quantification of cytokine levels in cell culture supernatants from macrophages was assessed. **(A)** Levels of pro-inflammatory cytokine IL6 (pg/mL). METs significantly increased IL6 production compared to Control (*p < 0.05 vs. Control). **(B)** Levels of anti-inflammatory cytokine IL10 (pg/mL). Significant changes in IL10 levels were observed with co-treatment of OP18 (#p < 0.05 vs. METs). The data is representative of two biologically independent experiments. Data are presented as mean ± SEM. Statistical significance was determined by one-way ANOVA with *post-hoc* Tukey test.

## Discussion

In this study, we report compelling evidence that the novel opsonic peptide OP18 effectively binds and enhances the clearance of radiation-induced METs. Our findings demonstrate that OP18 facilitates METs uptake through an α_v_β_3_-integrin mediated endocytic pathway, leading to their subsequent trafficking via the endo-lysosomal compartment. This accelerated clearance of METs by macrophages resulted in significant alleviation of mitochondrial stress, suppression of NF-κB activation, and a reduction in the release of pro-inflammatory cytokines, collectively mitigating METs-induced inflammation (**Figure 7**). As we reported previously, extracellular DNA was identified not only by SYTOX Orange staining but also by MPO staining in the same setting, indicating the presence of both elements to confirm METs (13). The DNA contains highly citrullinated histones, which are used as surrogate evidence of extracellular trap formation. In this study, we examined the coexistence of SYTOX Orange labeling and citrullinated histone H3 in the DNA prepared from irradiated macrophages.

**Figure 7:**
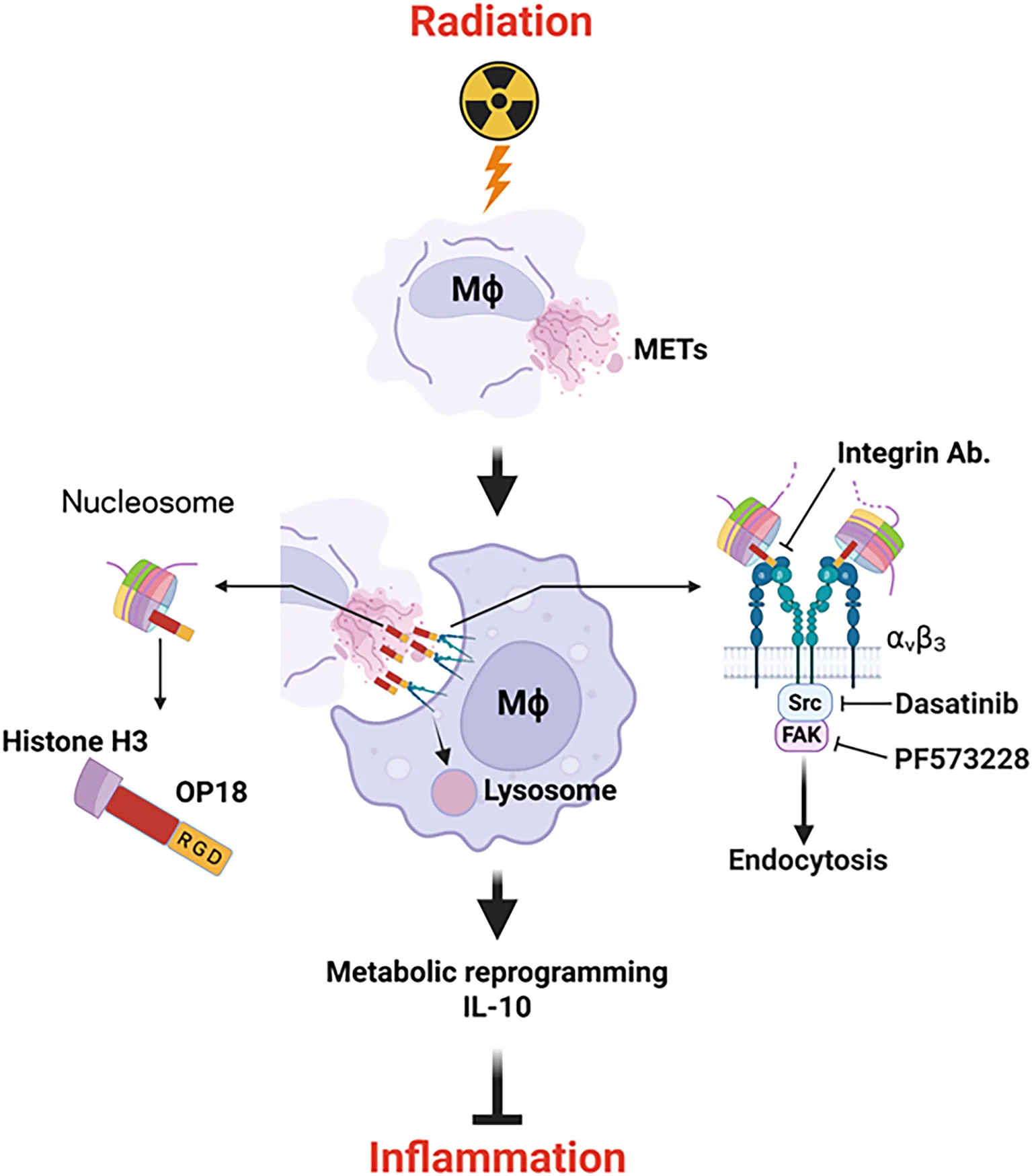
Graphic summary: Proposed mechanism of OP18-mediated METs clearance and inflammation reduction. Schematic illustration summarizing the hypothesized mechanism of action for OP18. OP18 enhances the uptake of macrophage extracellular traps (METs) by macrophages, a process mediated by α_v_β_3_-integrin. This enhanced uptake leads to accelerated clearance of METs via endo-lysosomal trafficking. Consequently, this leads to an alleviation of metabolic burden (e.g., mitochondrial stress) and a reduction in the inflammatory response, thereby mitigating METs-induced inflammation. Created in BioRender. Jin, V. (2026) https://BioRender.com/v5ke4m6.

A central finding of our work is the critical role of α_v_β_3_-integrin in OP18-mediated METs uptake. OP18 enhances the co-localization of METs with α_v_β_3_-integrin, and the inhibition of α_v_β_3_-integrin significantly reverses its capacity to promote METs clearance. This suggests that OP18 may act as a “MET-opsonic” molecule, promoting the interaction between METs and the integrin receptor on the macrophage surface, thereby facilitating endocytosis. Once internalized, OP18 directs METs towards LAMP1 and CD63-positive endo-lysosomal compartments, ensuring their efficient degradation rather than mere sequestration. Importantly, these mechanisms were observed not only in radiation-exposed macrophages but also in normal macrophages, broadening the applicability of OP18 beyond the context of radiation injury and suggesting its general utility in contexts of METs accumulation.

The effective clearance of METs by OP18 yields significant cellular benefits. Firstly, we observed a marked metabolic restoration. Persistent METs in the extracellular environment contribute to cellular burden (28), leading to mitochondrial dysfunction characterized by decreased ATP production, maximal respiration, and basal respiration. The ability of OP18 to clear these METs significantly reverses this mitochondrial damage, restoring metabolic homeostasis and energy production. Secondly, the enhanced clearance of METs translates into potent anti-inflammatory effects. METs are known activators of intracellular signaling pathways that drive inflammation. Our data shows that OP18 suppresses METs-induced NF-κB activation and its nuclear translocation. Given that NF-κB is a central mediator of pro-inflammatory gene expression, its inhibition by OP18 is critical for alleviating inflammatory responses. This suppression of NF-κB activation consequently leads to a reduction in the release of pro-inflammatory cytokines, specifically IL-6, further underscoring anti-inflammatory potential of OP18. Our data also revealed elevated levels of IL-10 released from MET-engulfed macrophages, which could explain the phenomenon of efferocytosis, a process where studies have demonstrated increased IL-10 release by efferocytotic macrophages due to their skewing towards an immune-suppressive state (29, 30). Future studies exploring the status and impact of IL-10-specific transcription factors hold great potential.

The implications of these findings are particularly significant for managing radiation-induced inflammation. Radiation exposure is known to trigger sterile inflammatory responses, often involving the generation of DAMPs and extracellular traps (8, 12, 19). By specifically targeting and clearing METs, which are likely key components of this inflammatory cascade, OP18 presents a novel and targeted therapeutic strategy. Unlike broad-spectrum anti-inflammatory drugs (14, 16), OP18 addresses a root cause of inflammation—the persistent presence of DAMPs (20, 21) and METs. This targeted approach holds potential for preventing chronic inflammation and associated pathologies that frequently follow radiation exposure in various clinical settings.

Our approach with OP18 diverges from general anti-inflammatory drugs by specifically focusing on the removal of pathogenic METs rather than broadly suppressing inflammatory mediators. While many therapies target downstream inflammatory signaling, OP18 aims to eliminate a potent upstream activator of inflammation, offering a more nuanced and potentially less immunosuppressive strategy. The accumulation of extracellular traps (ETs), including METs, is increasingly recognized as a pathological driver in numerous inflammatory and autoimmune conditions, such as sepsis, lupus, and cardiovascular diseases (28). Our findings align with a growing body of literature highlighting the therapeutic potential of strategies that promote ET clearance (16), positioning OP18 as a promising candidate within this emerging targeted therapeutic landscape.

While our *in vitro* data provide strong evidence for the efficacy and mechanism of OP18, a limitation is the need for validation in complex biological systems. The current study was primarily conducted *in vitro*, necessitating subsequent *in vivo* studies to confirm its therapeutic potential in animal models of radiation injury. While we have established the involvement of α_v_β_3_-integrin, due to the complexity of METs, the precise molecular interaction, including specific binding sites and affinity of OP18 with METs and α_v_β_3_-integrin, remains to be fully elucidated. Building upon these *in vitro* findings, future research will focus on several key areas. Comprehensive *in vivo* efficacy studies are crucial to evaluate OP18’s therapeutic potential in relevant animal models of radiation-induced complications. Mechanistically, further studies will investigate specific intracellular signaling pathways, beyond NF-κB, that are influenced by METs and OP18. Additionally, exploring the impact of OP18 on macrophage polarization, particularly its ability to modulate the M1/M2 balance in response to radiation and METs, could provide deeper insights into its immunomodulatory effects. While the uptake experiments used irradiated macrophages, we wanted to test the effect of OP18 in mitochondrial respiration, NF- κB, and cytokine assay in macrophages. Due to the compound effect of both radiation and METs to macrophages it was technically hard to dissect one from the other. Therefore, we focused on the mechanistic role of OP18 mediated METs clearance in non-irradiated macrophages. This data is effects on responses to radiation-induced METs rather than reversal of radiation-induced macrophage dysfunction. Further mechanistic study would explain direct effect of OP18 on the radiation induced dysfunction in the future. Lastly, given the broad implication of ETs in disease, investigating the potential of OP18 in other METs-driven inflammatory diseases beyond radiation injury warrants exploration.

In conclusion, OP18 enhances METs clearance and subsequently reduces inflammation in macrophages. We showed that the radiation-induced METs are being taken up by macrophages, with OP18 enhancing this uptake process. We identified the role of integrins in this uptake and subsequent trafficking of METs into the endo-lysosomal pathway for degradation. The consequence of enhanced METs clearance is shown to be an alleviation of metabolic burden as indicated by improved mitochondrial function and a reduction in the inflammatory response, ultimately leading to reduced radiation-induced inflammation and potentially other inflammatory conditions driven by extracellular traps.

## Data Availability

The original contributions presented in the study are included in the article/**Supplementary Material**. Further inquiries can be directed to the corresponding authors.
