# Supplementary material for: An opsonic peptide promotes radiation-induced macrophage extracellular trap clearance to alleviate inflammation
Source: Front Immunol. 2026 Oct 12;17:1970645. doi: 10.3389/fimmu.2026.1970645 (PMC13696247; doi:10.3389/fimmu.2026.1970645)
Supplement: Supplementary file 1 [file DataSheet1.pdf]

Supplementary Figure 1

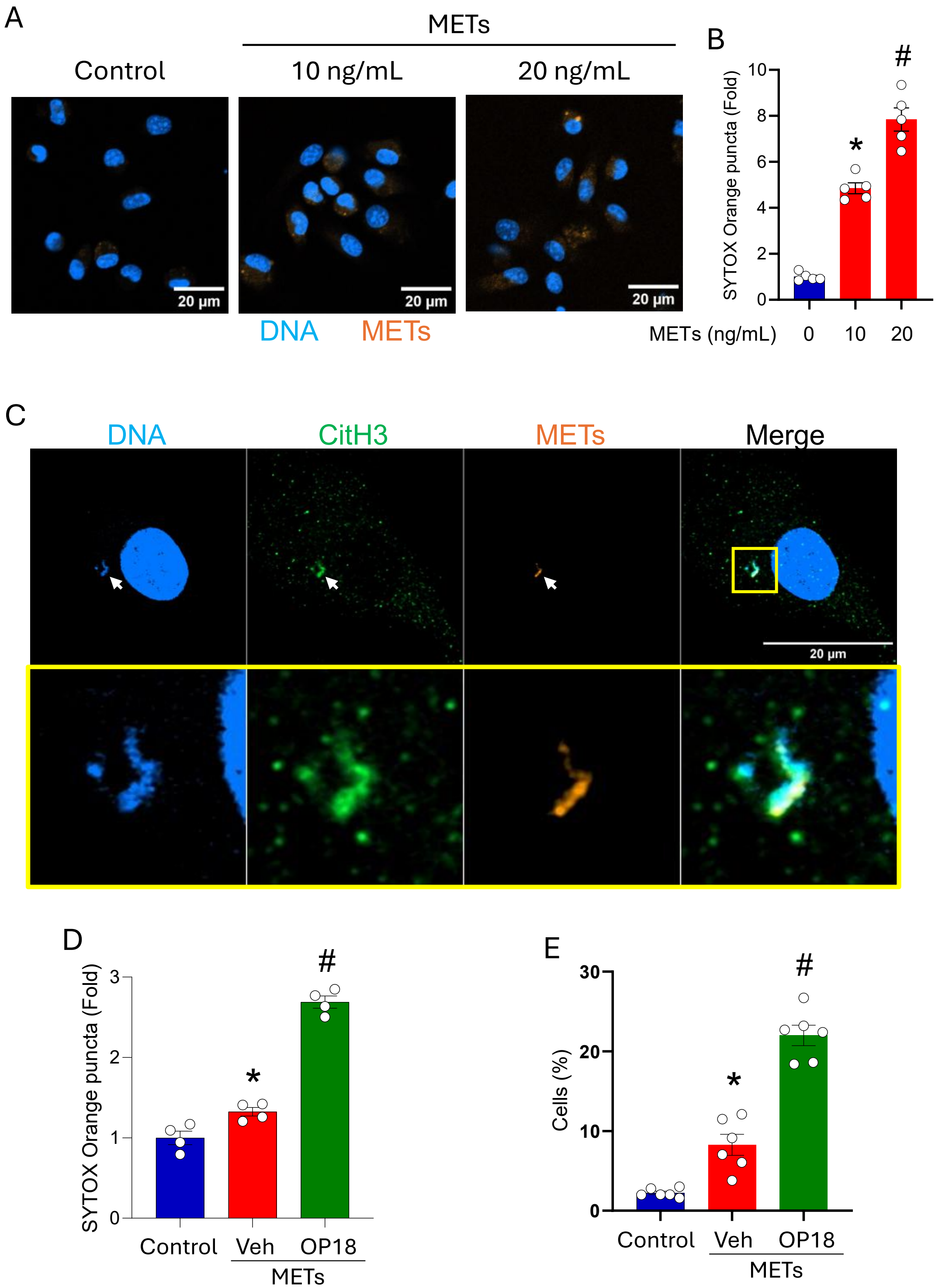

Supplementary Figure 1. OP18 enhances extracellular trap clearance by normal (non-irradiated) macrophages

(A) Representative confocal images of DNA (blue) and METs (SYTOX Orange, orange) in macrophages. SYTOX Orange stained METs was treated as indicated and Control group was not treated with METs DNA. **(B)** Fold increase in both 10 ng/mL and 20 ng/mL METs showed dose-dependent response (*$p < 0.05$ vs. PBS; #$p < 0.05$ vs. 10 ng METs). **(C)** Representative confocal images of DNA (blue), CitH3 (METs component, green), and METs (SYTOX Orange, orange) in macrophages treated with 20 ng METs. **(D)** Fold change in SYTOX Orange-positive puncta per cell for PBS, METs, and OP18 + METs. OP18 significantly enhanced METs uptake compared to METs alone (#$p < 0.05$ vs. METs). Data are presented as mean ± SEM. Statistical significance was determined by one-way ANOVA with post-hoc Tukey test. **(E)** Percentage of cells positive for METs in Control, METs (20 ng/mL), and METs + OP18 (2 µg/mL) groups were assessed by flow cytometry. METs significantly increased the percentage of positive cells (*$p < 0.05$ vs. Control), and OP18 significantly further increased this percentage (#$p < 0.05$ vs. METs). Data are presented as mean ± SEM. Statistical significance was determined by one-way ANOVA with post-hoc Tukey test.

Supplementary Figure 2

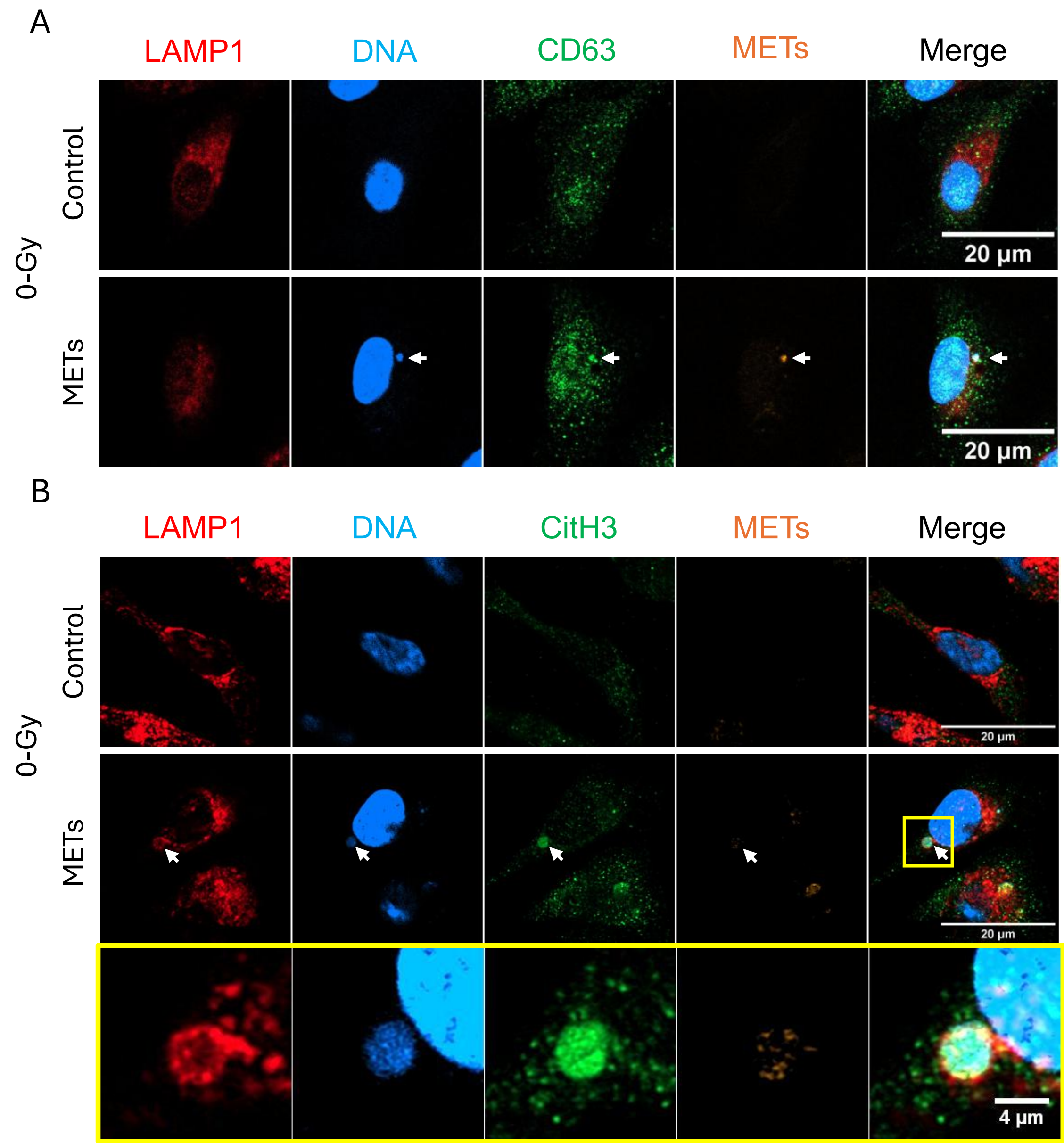

Supplementary Figure 2. Clearance of macrophage extracellular traps (METs) via endo-lysosomal trafficking in normal (non-irradiated) macrophages.

Confocal microscopy images illustrate the endo-lysosomal trafficking of macrophage extracellular traps (METs) in normal (non-irradiated) macrophages. Macrophages were treated with exogenous METs (20 ng/mL), and subsequently fixed and stained. **(A)** Representative images showing co-localization of DNA (blue), CD63 (endosomal/lysosomal marker, green), and

SYTOX Orange (orange). **(B)** Representative images showing co-localization of LAMP1 (lysosomal marker, red), DNA (blue), CitH3 (METs component, green), and SYTOX Orange (METs DNA, orange) in normal macrophages treated with Control or METs.
